# Medical expenditure for esophageal cancer in China: a 10-year multicenter retrospective survey (2002–2011)

**DOI:** 10.1186/s40880-017-0242-3

**Published:** 2017-09-07

**Authors:** Lan-Wei Guo, Hui-Yao Huang, Ju-Fang Shi, Li-Hong Lv, Ya-Na Bai, A-Yan Mao, Xian-Zhen Liao, Guo-Xiang Liu, Jian-Song Ren, Xiao-Jie Sun, Xin-Yu Zhu, Jin-Yi Zhou, Ji-Yong Gong, Qi Zhou, Lin Zhu, Yu-Qin Liu, Bing-Bing Song, Ling-Bin Du, Xiao-Jing Xing, Pei-An Lou, Xiao-Hua Sun, Xiao Qi, Shou-Ling Wu, Rong Cao, Li Lan, Ying Ren, Kai Zhang, Jie He, Jian-Gong Zhang, Min Dai, on behalf of the Health Economic Evaluation Working Group, Cancer Screening Program in Urban China (CanSPUC)

**Affiliations:** 1 Department of Cancer Epidemiology, Henan Office for Cancer Control and Research, The Affiliated Cancer Hospital of Zhengzhou University, Henan Cancer Hospital, No. 127 Dongming Road, Zhengzhou, 450008 Henan P. R. China; 2 Program Office for Cancer Screening in Urban China, Cancer Hospital, Chinese Academy of Medical Sciences (CAMS), Peking Union Medical College, National Cancer Center of China, No. 17 Panjiayuannanli, Chaoyang District, Beijing, 100021 P. R. China; 3 Department of Human Resources, The Affiliated Cancer Hospital of Zhengzhou University, Henan Cancer Hospital, Zhengzhou, 450008 Henan P. R. China; 4 Institute of Epidemiology and Health Statistics, Lanzhou University, Lanzhou, 730000 Gansu P. R. China; 5 Public Health Information Research Office, Institute of Medical Information, Chinese Academy of Medical Sciences (CAMS), Beijing, 100020 P. R. China; 6 Hunan Office for Cancer Control and Research, Hunan Provincial Cancer Hospital, Changsha, 410006 Hunan P. R. China; 7 Department of Health Economics, School of Health Management, Harbin Medical University, Harbin, 150081 Heilongjiang P. R. China; 8 Center for Health Management and Policy, Key Lab of Health Economics and Policy, Shandong University, Jinan, 250012 Shandong P. R. China; 9 Institute of Chronic Non-Communicable Diseases Prevention and Control, Jiangsu Provincial Center for Disease Control and Prevention, Nanjing, 210009 Jiangsu P. R. China; 10 Science and Education Department of Public Health Division, Shandong Tumor Hospital, Jinan, 250117 Shandong P. R. China; 11 Chongqing Office for Cancer Control and Research, Chongqing Cancer Hospital, Chongqing, 400030 P. R. China; 12 Teaching and Research Department, Affiliated Cancer Hospital of Xinjiang Medical University, Ürümqi, 830011 Xinjiang P. R. China; 13 Cancer Epidemiology Research Center, Gansu Provincial Cancer Hospital, Lanzhou, 730050 Gansu P. R. China; 14 Heilongjiang Office for Cancer Control and Research, Affiliated Cancer Hospital of Harbin Medical University, Harbin, 150081 Heilongjiang P. R. China; 15 Zhejiang Office for Cancer Control and Research, Zhejiang Cancer Hospital, Hangzhou, 310022 Zhejiang P. R. China; 16 Liaoning Office for Cancer Control and Research, Liaoning Cancer Hospital & Institute, Shenyang, 110042 Liaoning P. R. China; 17 Department of Control and Prevention of Chronic Non-Communicable Diseases, Xuzhou Center for Disease Control and Prevention, Xuzhou, 221006 Jiangsu P. R. China; 18 Ningbo Clinical Cancer Prevention Guidance Center, Ningbo NO. 2 Hospital, Ningbo, 315010 Zhejiang P. R. China; 19 Department of Occupational Medicine, Tangshan People’s Hospital, Tangshan, 063001 Hebei P. R. China; 20 Health Department of Kailuan Group, Kailuan General Hospital, Tangshan, 063000 Hebei P. R. China; 21 Department of Health Policy and Economic Research, Guangdong Provincial Institute of Public Health, Guangzhou, 511430 Guangdong P. R. China; 22 Institute of Chronic Disease Prevention and Control, Harbin Center for Disease Control and Prevention, Harbin, 150056 Heilongjiang P. R. China; 23 Urban Office of Cancer Early Detection and Treatment, Tieling Central Hospital, Tieling, 112000 Liaoning P. R. China

**Keywords:** Esophageal neoplasms, Medical expenditure, Diagnosis and treatment, China

## Abstract

**Background:**

Esophageal cancer is associated with substantial disease burden in China, and data on the economic burden are fundamental for setting priorities in cancer interventions. The medical expenditure for the diagnosis and treatment of esophageal cancer in China has not been fully quantified. This study aimed to examine the medical expenditure of Chinese patients with esophageal cancer and the associated trends.

**Methods:**

From 2012 to 2014, a hospital-based multicenter retrospective survey was conducted in 37 hospitals in 13 provinces/municipalities across China as a part of the Cancer Screening Program of Urban China. For each esophageal cancer patient diagnosed between 2002 and 2011, clinical information and expense data were extracted by using structured questionnaires. All expense data were reported in Chinese Yuan (CNY; 1 CNY = 0.155 USD) based on the 2011 value and inflated using the year-specific health care consumer price index for China.

**Results:**

A total of 14,967 esophageal cancer patients were included in the analysis. It was estimated that the overall average expenditure per patient was 38,666 CNY, and an average annual increase of 6.27% was observed from 2002 (25,111 CNY) to 2011 (46,124 CNY). The average expenditures were 34,460 CNY for stage I, 39,302 CNY for stage II, 40,353 CNY for stage III, and 37,432 CNY for stage IV diseases (*P* < 0.01). The expenditure also differed by the therapy type, which was 38,492 CNY for surgery, 27,933 CNY for radiotherapy, and 27,805 CNY for chemotherapy (*P* < 0.05). Drugs contributed to 45.02% of the overall expenditure.

**Conclusions:**

These conservative estimates suggested that medical expenditures for esophageal cancer in China substantially increased in the last 10 years, treatment for early-stage esophageal cancer costs less than that for advanced cases, and spending on drugs continued to account for a considerable proportion of the overall expenditure.

## Background

Esophageal cancer is the eighth most common cancer worldwide with estimates of 455,784 new cases and 400,156 deaths in 2012 [1]. The incidence varies in different physiographic regions, nations, and races. China had the highest number of new esophageal cancer cases (more than 223,000) worldwide in 2012 [2]. The number of new esophageal cancer cases in China will increase to 433,783 by the year 2035 [1]. In the meantime, in China, early detection of esophageal cancer has increased, while mortality has decreased [3, 4]. As the survival for esophageal cancer patients is prolonging and the average cost of esophageal cancer treatments is increasing, the economic burden of patients has continued to grow. Therefore, the need for prevention strategies is urgent, and the implementation of effective measures should be based on evidence from health economic analyses.

The Cancer Screening Program in Urban China (CanSPUC), which was supported by the National Health and Family Planning Commission (NHFPC) in China, has been a crucial cancer prevention initiative that began in August 2012 [5]. The primary objective of the CanSPUC was to explore an appropriate implementation approach for screening of population at high risk and early diagnosis of major cancers (including lung, female breast, colorectal, esophageal, gastric, and liver cancers) in urban populations in China, promoting the use of mature screening and early diagnosis technology for common cancers to reduce mortalities. Taking funding and local conditions into account, the program covered 13 provinces/municipalities in China [including eastern (Beijing, Hebei, Liaoning, Jiangsu, Zhejiang, Shandong, and Guangdong), central (Heilongjiang, Henan, and Hunan), and western regions (Chongqing, Gansu, and Xinjiang)] [6] by 2014 in the first stage and targets urban residents who were between 40 and 69 years of age. As a part of the CanSPUC, the main content of the health economic evaluation includes expenditure and economic burden assessment, quality of life and health utility surveys, multi-angle project sustainability assessment, clinical diagnosis and treatment, and cost-effective model-based analysis. The goal is to provide the scientific evidence for expanding cancer screening and health decision-making in early detection and treatment programs for the Chinese population.

Few studies have examined the medical expenditure of Chinese patients with esophageal cancer and their associated trends [7]. In this study, we used data collected from a retrospective survey conducted by the CanSPUC to estimate the medical expenditures for esophageal cancer during the past 10 years, from 2002 to 2011, and to analyze the time trends over the long term.

## Methods

### Data sources

This survey was approved by the Ethics Committee of the Cancer Hospital, Chinese Academy of Medical Sciences (CAMS) and was conducted from 2012 to 2014 in 13 pilot provinces/municipalities in China. Basic information, clinical diagnosis, and medical expense information of patients with esophageal cancer (ICD10: C15), discharged between 2002 and 2011, were collected from historical medical records of 37 hospitals in these provinces/municipalities. Hospitals in China are organized according to a 3-tier system that recognizes a hospital’s ability to provide medical care and medical education and conduct medical research [6]. Based on this system, hospitals are designated as primary, secondary, or tertiary institutions. Furthermore, based on the level of service provision, size, medical technology, medical equipment, management and medical care quality, these 3 grades are further subdivided into 3 subsidiary levels A, B, and C, resulting in a total of 9 levels. In addition, one special level, 3A, is reserved for the most specialized hospitals. Among the information extracted, medical expense information, including the registration fee, ward bed fee, diagnosis fee, inspection fee, treatment fee, surgery fee, laboratory fee, nursing fee, drug fee, and other fees, refers to the medical costs for all physician visits from the first to the last treatments for esophageal cancer in the same hospital. This information was collected by a standard questionnaire or exported through the hospital electronic medical record (EMR) system, if possible. The database we created is a primary data source for this study that provides information on the diagnosis, treatment, and medical care costs for patients with esophageal cancer.

### Inclusion and exclusion criteria

Esophageal cancer patients were enrolled in the study if they met the following criteria: (1) diagnosed with esophageal cancer (patients with advanced metastatic cancer should have esophageal cancer as the primary tumor); (2) previously hospitalized in one of the hospitals participating in the project, and the main treatment expenses occurred at that hospital; (3) basic personal and medical expense information was available, and clinical information (diagnosis, treatment, and pathologic diagnostics) was complete; and (4) the last hospital discharge was between Jan 1, 2002 and Dec 31, 2011.

The exclusion criteria were as follows: (1) diagnosed with two or more cancers; (2) not treated in the participating hospitals; (3) basic personal or medical expense information was not available, or the clinical information such as the time for the last discharge from the hospital, date of diagnosis, and overall medical expenditure was not complete; or (4) the last hospital discharge was not between Jan 1, 2002 and Dec 31, 2011.

### Sample size

For each province/municipality, at least 120 esophageal cancer cases were selected in each year during 2002–2011, and the expected total sample size from 13 provinces/municipalities was 15,600. In this survey, the proportion of eligible cases selected in each year in each city involved in the present survey was required to stay balanced for each clinical stage (I–IV) and gender, resulting in a sufficient number of cases for subgroup analysis.

### Quality control procedures

First, the questionnaire design was confirmed by an expert reasoning committee. Second, esophageal cancer cases were selected according to the standard inclusion criteria. The information was collected via standard questionnaire by trained interviewers or exported through the hospital EMR system, if possible. Third, multi-level quality control was applied. The self-test was used when collecting information, and quality control staff then checked it again. Finally, a data administrator from the National Cancer Center of China would perform verification again after receiving the data. Data with mistakes or missing information was returned to the corresponding province/municipality.

### Statistical analysis

A database was established using EpiData *v* 3.1 software (EpiData Association, Odense, Denmark). Data were entered at the provincial level; one person entered the data twice using the “Prepare Double Entry Verification” function in the EpiData *v* 3.1 software. SAS statistical software, version 9.3 (SAS Institute, Cary/NC, USA), was used for all statistical analysis. A two-tailed *P* < 0.05 was considered statistically significant.

All expense data were reported in Chinese Yuan (CNY) based on the 2011 value, which was inflated using the year-specific personal health care consumer price index (CPI) of China. Medical expenditure per patient was defined as the total expenditure for all clinical visits per patient, and the reporting deadline for the expenses was the year corresponding to the last hospital discharge.

Medical expenditure per patient was described as the mean ± standard deviation (SD) and compared using the 2-sample Student *t* test or ANOVA test after logarithm transition. The time trend of medical expenditures from 2002 to 2011 was described, and the Average Tempo was used to calculate an annual average increasing rate of medical expenditures.

## Results

### Summary of the survey sites and hospitals

Our study was conducted in 13 provinces/municipalities in China. The population size and gross domestic product (GDP) per capita in 2011 are presented in Table 1. Thirty-seven hospitals in 22 cities participated in the study, which included 22 general hospitals and 15 specialized hospitals.

**Table 1:** Summary of the survey sites and hospitals in 13 provinces/municipalities in China

Province/municipality	General information of the province/municipality		Specific information on survey sites and involved hospitals			
	Population size in 2011^a^ (×10,000)	GDP per capita in 2011^a^ (CNY)	No. of cities involved	No. of hospitals involved	No. (level) of general hospitals involved^b^	No. (level) of specialized hospitals involved^b^
Eastern region						
Beijing	2019	81,658	1 (Beijing)	5	3 (3A for 2, 3A less for 1)	2 (3A for both)
Hebei	7241	33,969	1 (Tangshan)	2	2 (3A for both)	0
Liaoning	4383	50,760	2 (Shenyang, Tieling)	2	1 (3A)	1 (3A)
Jiangsu	7899	62,290	2 (Nantong, Xuzhou)	3	1 (3A less)	2 (3A for both)
Zhejiang	5463	59,249	2 (Hangzhou, Ningbo)	2	1 (3A)	1 (3A)
Shandong	9637	47,335	1 (Jinan)	1	0	1 (3A)
Guangdong	10,505	50,807	5 (Guangzhou, Shenzhen, Dongguan, Foshan, and Zhongshan)	6	6 (3A for all)	0
Central region						
Heilongjiang	3834	32,819	2 (Harbin, Daqing)	4	2 (3A for both)	2 (3A for both)
Henan	9388	28,661	1 (Zhengzhou)	1	0	1 (3A)
Hunan	6596	29,880	1 (Changsha)	1	0	1 (3A)
Western region						
Chongqing	2919	34,500	1 (Chongqing)	1	0	1 (3A)
Gansu	2564	19,595	2 (Lanzhou, Jinchang)	8	6 (3A less for all)	2 (3A for 1, 3A less for 1)
Xinjiang	2209	30,087	1 (Urumqi)	1	0	1 (3A)
Overall	–	–	22	37	22	15

^a^Based on the China Statistical Yearbook 2012, http://www.stats.gov.cn/tjsj/ndsj/2012/indexch.htm

^b^Hospitals in China are organized according to a 3-tier system that recognizes a hospital’s ability to provide medical care and medical education and conduct medical research. Based on this, hospitals are designated as primary, secondary, or tertiary institutions. Furthermore, based on the level of service provision, size, medical technology, medical equipment, and management and medical care quality, these 3 grades are further subdivided into 3 subsidiary levels A, B, and C, resulting in a total of 9 levels. In addition, one special level, 3A, is reserved for the most specialized hospitals

### Characteristics of included esophageal cancer cases

A total of 14,967 esophageal cancer patients (males accounted for 78.29%), with an average diagnosis age of 60.93 ± 9.90 years (median: 61 years; percentile 25-percentile 75 [P25–P75]: 54–68 years), were included in the analysis (Table 2). Of these, 47.12% were from the eastern region of China, 77.08% were from specialized hospitals, and 91.88% were from 3A hospitals. Stage I esophageal cancer patients accounted for 7.56% of the sample, and esophageal squamous cell carcinoma (ESCC) was the most common pathologic type (85.06%). The median total number of admissions/visits was 1 (percentile 5-percentile 95 [P5–P95]: 1–5), and the patients who only visited the hospital once accounted for 63.90% of the sample. The included patients spent a median of 28 hospitalization days receiving cancer treatment (range 1–507 days; P25–P75: 18–50 days). The main therapy types were surgery alone (34.75%), surgery and chemotherapy (20.17%), radiotherapy alone (13.33%), and chemotherapy alone (11.36%).

**Table 2:** Characteristics of the 14,967 selected esophageal cancer patients, 2002–2011

Variable	Number of patients [cases (%)]
Region	
Eastern	7053 (47.12)
Central	4287 (28.64)
Western	3627 (24.23)
Hospital type	
General	3430 (22.92)
Specialized	11,537 (77.08)
Hospital level	
3A	13,752 (91.88)
3A less	1215 (8.12)
Gender	
Male	11,717 (78.29)
Female	3250 (21.71)
Age at diagnosis (years)	
<45	666 (4.45)
45–54	3252 (21.73)
55–64	5683 (37.97)
≥65	5366 (35.85)
Pathologic type^a^	
Squamous cell carcinoma	12,401 (85.06)
Others	2178 (14.94)
Clinical stage	
I	1131 (7.56)
II	5186 (34.65)
III	4695 (31.37)
IV	2880 (19.24)
Not reported	1075 (7.18)
Morphologic verification	13,332 (89.10)
Number of clinical visits per case	
1	9564 (63.90)
2	2725 (18.21)
3	1176 (7.86)
4+	1502 (10.04)
Type of therapy^b^	
Surgery	5168 (34.75)
Surgery and chemotherapy	3000 (20.17)
Radiotherapy	1982 (13.33)
Chemotherapy	1690 (11.36)
Radiotherapy and chemotherapy	1310 (8.81)
Surgery and radiotherapy	386 (2.60)
Palliative care	928 (6.24)
Others	408 (2.74)
Comorbidities	3877 (25.9)
Complications	1235 (8.25)

^a^The data for 388 patients were missing

^b^The data for 95 patients were missing

### Medical expenditure for esophageal cancer diagnosis and treatment per patient

The average expenditure per patient increased with the clinical stage (*P* < 0.001). The expenditures also differed according to the therapy type, region, hospital level and category, number of clinical visits, gender, age at diagnosis, and pathologic type (all *P* < 0.05). Additionally, since recent data are more applicable to current and future use, the expense data for the last 3 years (2009–2011) were also analyzed (Table 3). The average expenditures during 2009–2011 were higher than those during 2002–2011 for all subgroups.

**Table 3:** Subgroup analysis of medical expenditures for esophageal cancer diagnosis and treatment per patient

Variable	Expenditure per patient during 2002–2011 (CNY)	Statistics^a^	*P*	Expenditure per patient during 2009–2011 (CNY)	Statistics^a^	*P*
Overall	38,666 (38,178–39,155)			45,319 (44,425–46,213)		
Region		649.44	<0.001		306.1	<0.001
Eastern	41,943 (41,202–42,684)			47,213 (46,021–48,406)		
Central	40,410 (39,646–41,173)			52,765 (51,100–54,430)		
Western	30,234 (29,200–31,269)			32,555 (30,585–34,526)		
Hospital type		−6.75	<0.001		−1.20	0.228
General hospital	36,684 (35,663–37,704)			45,706 (43,883–47,529)		
Specialized hospital	39,256 (38,700–39,812)			45,182 (44,157–46,207)		
Hospital level		25.60	<0.001		13.26	<0.001
3A	40,134 (39,625–40,643)			46,751 (45,824–47,677)		
3A less	22,052 (20,621–23,483)			26,409 (23,612–29,206)		
Number of clinical visits per patient		889.53	<0.001		204.28	< 0.001
1	29,089 (28,651–29,528)			34,858 (33,981–35,735)		
2	46,633 (45,530–47,737)			53,959 (52,002–55,917)		
3	59,441 (57,524–61,357)			60,407 (57,497–63,317)		
4+	68,928 (66,774–71,082)			69,055 (65,749–72,360)		
Gender		5.64	<0.001		1.84	0.066
Male	39,521 (38,961–40,082)			46,046 (45,017–47,074)		
Female	35,585 (34,605–36,564)			42,768 (40,976–44,561)		
Age at diagnosis (years)		8.65	<0.001		14.42	<0.001
<45	37,796 (35,539–40,054)			49,384 (43,380–55,388)		
45–54	39,237 (38,172–40,302)			50,732 (48,367–53,096)		
55–64	39,588 (38,792–40,384)			46,572 (45,139–48,006)		
≥65	37,452 (36,647–38,257)			41,741 (40,426–43,057)		
Pathologic type		23.95	<0.001		11.61	<0.001
Squamous cell carcinoma	40,711 (40,173–41,250)			48,242 (47,233–49,250)		
Others	30,216 (29,032–31,399)			35,576 (33,636–37,516)		
Clinical stage		55.51	<0.001		21.80	<0.001
I	34,460 (32,902–36,017)			40,000 (36,878–43,122)		
II	39,302 (38,526–40,079)			47,978 (46,381–49,575)		
III	40,353 (39,491–41,214)			44,962 (43,616–46,309)		
IV	37,432 (36,180–38,685)			42,972 (40,749–45,194)		
Type of therapy		758.66	<0.001		270.53	<0.001
Surgery	38,492 (37,890–39,094)			47,778 (46,548–49,008)		
Surgery and chemotherapy	51,503 (50,316–52,691)			63,843 (61,535–66,151)		
Radiotherapy	27,933 (26,787–29,079)			29,085 (27,471–30,699)		
Chemotherapy	27,805 (26,310–29,299)			35,671 (33,121–38,222)		
Radiotherapy and chemotherapy	53,907 (51,867–55,946)			57,234 (53,865–60,603)		
Surgery and radiotherapy	57,725 (55,041–60,409)			62,438 (58,708–66,167)		
Palliative care	13,846 (12,626–15,067)			15,627 (13,187–18,067)		
Others	23,057 (20,564–25,550)			21,821 (18,386–25,256)		

All expenditure data are presented as mean with 95% confidence interval in parentheses

*CNY* Chinese Yuan

^a^The two-sample Student *t* test after logarithm transition was used for binary classification variables, including hospital type, hospital level, gender, and pathologic type. The ANOVA test after logarithm transition was used for other multiple categorical variables, including region, number of clinical visits per patient, age at diagnosis, and clinical stage

The estimated overall average expenditure per patient was 38,666 CNY in 2002–2011 (Table 3), and more than 90% (36,173 CNY, 95% confidence interval [CI]: 35,721–36,624 CNY) was spent within 2 months before and 10 months after the diagnosis.

**Fig. 1:**
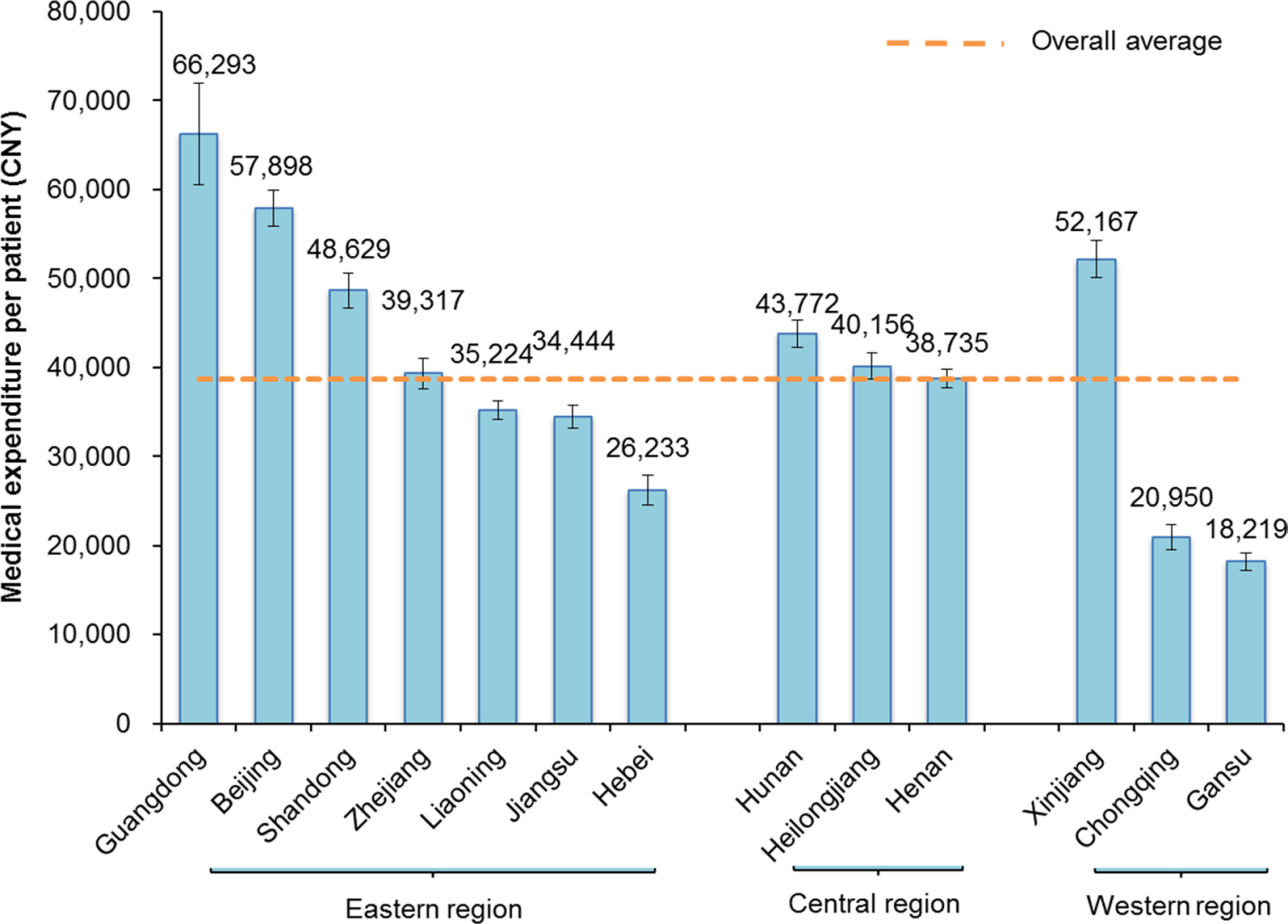
Medical expenditure for esophageal cancer diagnosis and treatment per patient in China, by province/municipality. China is divided into the eastern, central, and western regions. Guangdong, Beijing, Shandong, Zhejiang, Liaoning, Jiangsu, and Hebei belong to the eastern region of China; Hunan, Heilongjiang, and Henan belong to the central region of China; and Xinjiang, Chongqing, and Gansu belong to the western region of China. Each bar indicates the average expenditure with 95% confidence interval in parentheses

The average expenditure per patient differed among geographical regions and survey sites. Of all 13 provinces/municipalities, Guangdong, Beijing, and Xinjiang had the highest expenditures (more than 50,000 CNY per patient); Hebei, Chongqing, and Gansu had the lowest expenditures (less than 30,000 CNY per patient); and Zhejiang, Heilongjiang, and Henan had nearly average expenditures (Fig. 1).

### Time trend of medical expenditures and related factors

**Fig. 2:**
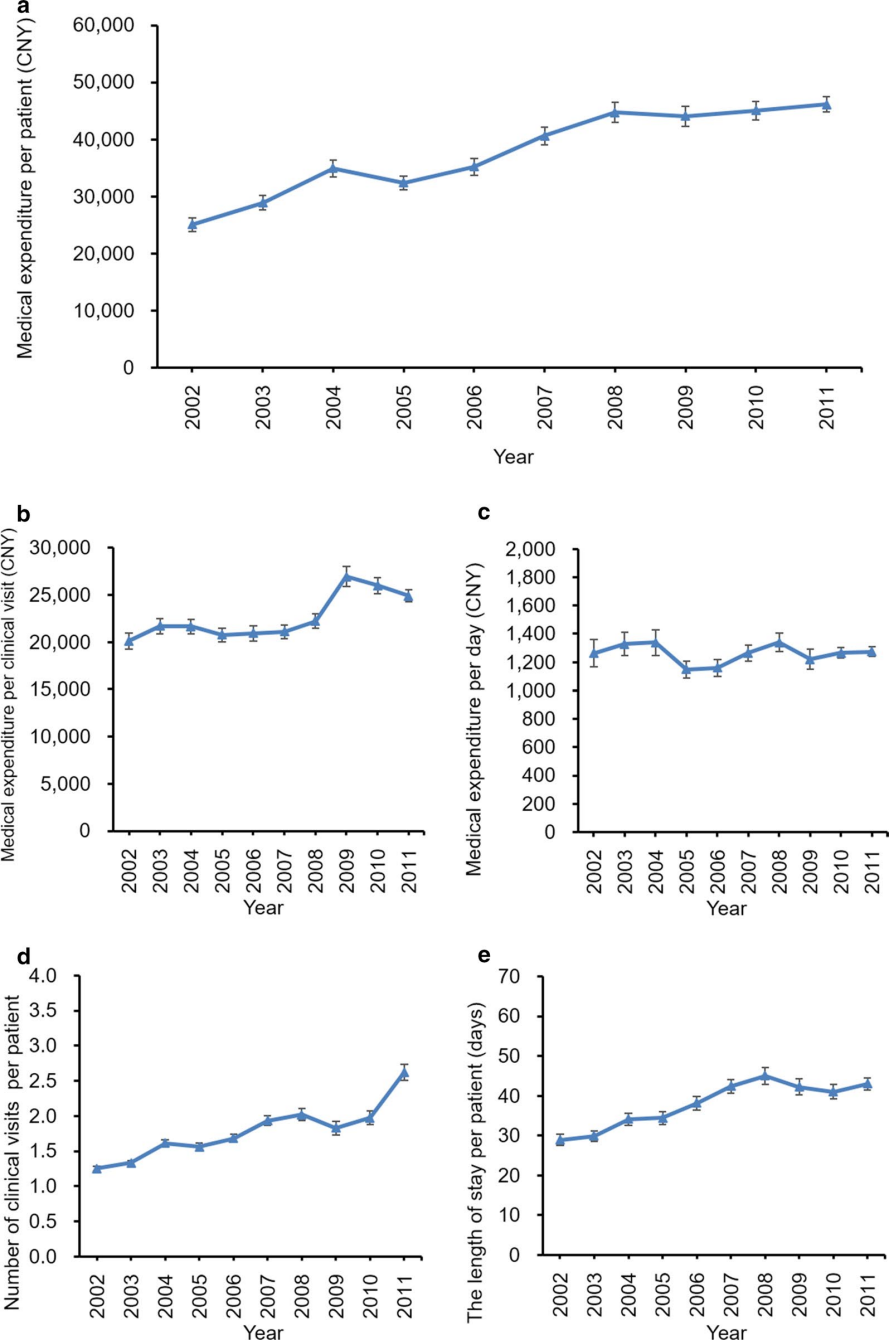
Time trend of medical expenditures and related factors for esophageal cancer diagnosis and treatment in China, 2002–2011. **a** Time trend of overall average medical expenditures per patient; **b** time trend of medical expenditures per clinical visit; **c**: time trend of daily average medical expenditure; **d** time trend of number of clinical visits per patient; **e** time trend of the length of hospitalization stay per patient

It was estimated that the overall average expenditure per patient increased by 6.27% per year from 25,111 CNY in 2002 to 46,124 CNY in 2011. A turning point was observed around 2009 (Fig. 2a); the average rates of increase before and after that point were 8.60% and 1.51%, indicating that the average tempo slowed down after 2009. The expenditure per clinical visit increased by 2.15% per year from 20,117 CNY in 2002 to 24,896 CNY in 2011 (Fig. 2b), and the daily average expenditure increased by 0.09% per year from 1262 CNY in 2002 to 1274 CNY in 2011 (Fig. 2c). The average number of clinical visits significantly increased from 1.25 in 2002 to 2.62 in 2011 (Fig. 2d). The length of hospitalization stay per patient significantly increased from 28.92 days in 2002 to 43.00 days in 2011, and the longest stay was 45.00 days in 2008 (Fig. 2e).

**Fig. 3:**
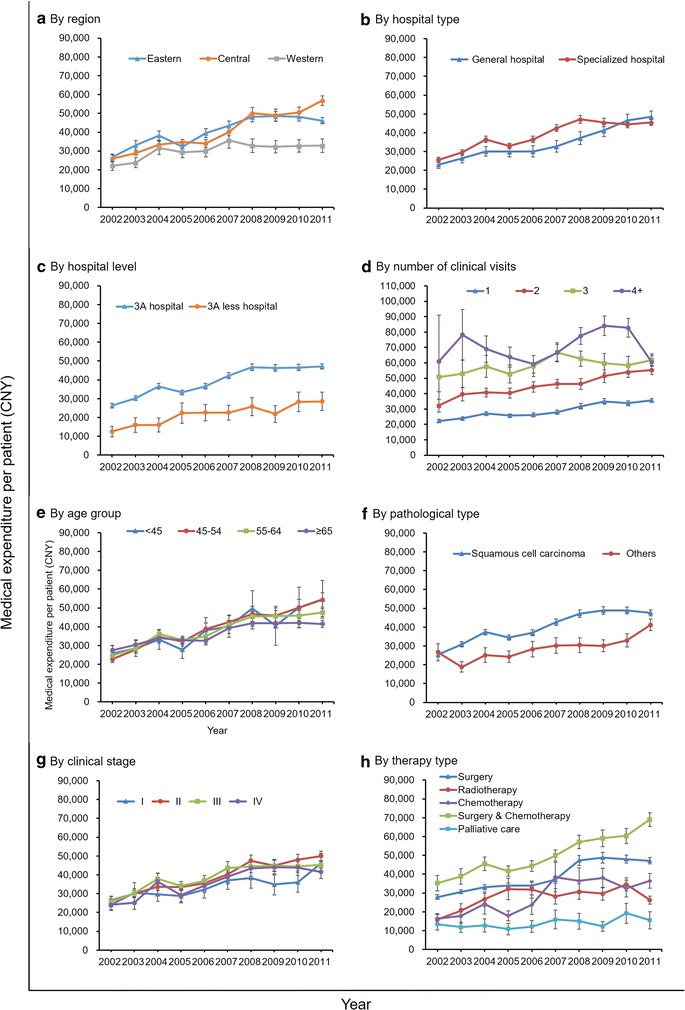
Subgroup analysis on time trend of medical expenditures for esophageal cancer diagnosis and treatment per patient in China, 2002–2011. **a** Time trend of medical expenditures stratified by region in China; **b** time trend of medical expenditures stratified by hospital type; **c** time trend of medical expenditures stratified by hospital level; **d** time trend of medical expenditures stratified by number of clinical visits per patient; **e** time trend of medical expenditures stratified by age group; **f** time trend of medical expenditures stratified by pathologic type; **g** time trend of medical expenditures stratified by clinical stage; **h** time trend of medical expenditures stratified by therapy type

The time trend of average expenditures differed according to geographical region, hospital type, hospital level, number of clinical visits, pathologic type, clinical stage, and therapy type (Fig. 3). The overall average expenditure per patient for the eastern and central regions of China increased faster than that for the western region of China, and the gap between those regions continued to widen (Fig. 3a). The overall average expenditure per patient for the central region of China after 2008 was higher than that for the eastern region of China, and the gap gradually widened. The overall average expenditure per patient for the central region of China in 2011 was 1.73 times that for the western region of China. Additionally, the gap between general hospitals and specialized hospitals gradually decreased (Fig. 3b), but the gap between 3A hospitals and 3A less hospitals increased (Fig. 3c). The overall average expenditure per patient for 3A hospitals was 1.65 times that for 3A less hospitals in 2011. The overall average expenditure per patient for stage I disease was much lower than those for stages II, III, and IV diseases, and the gap increased after 2008 (Fig. 3g). The overall average expenditure per patient for stages II, III, and IV diseases did not show a significant difference. Stratification by therapy type showed that the overall average expenditure per patient increased faster for the chemotherapy alone, surgery alone, and surgery combined chemotherapy subgroups, but increased gradually for the palliative care subgroup (Fig. 3h). Further details on the expenditure time trends are also presented by the number of clinical visits, age at diagnosis, and pathologic type (Fig. 3).

### The proportional breakdown of medical expenditures

**Fig. 4:**
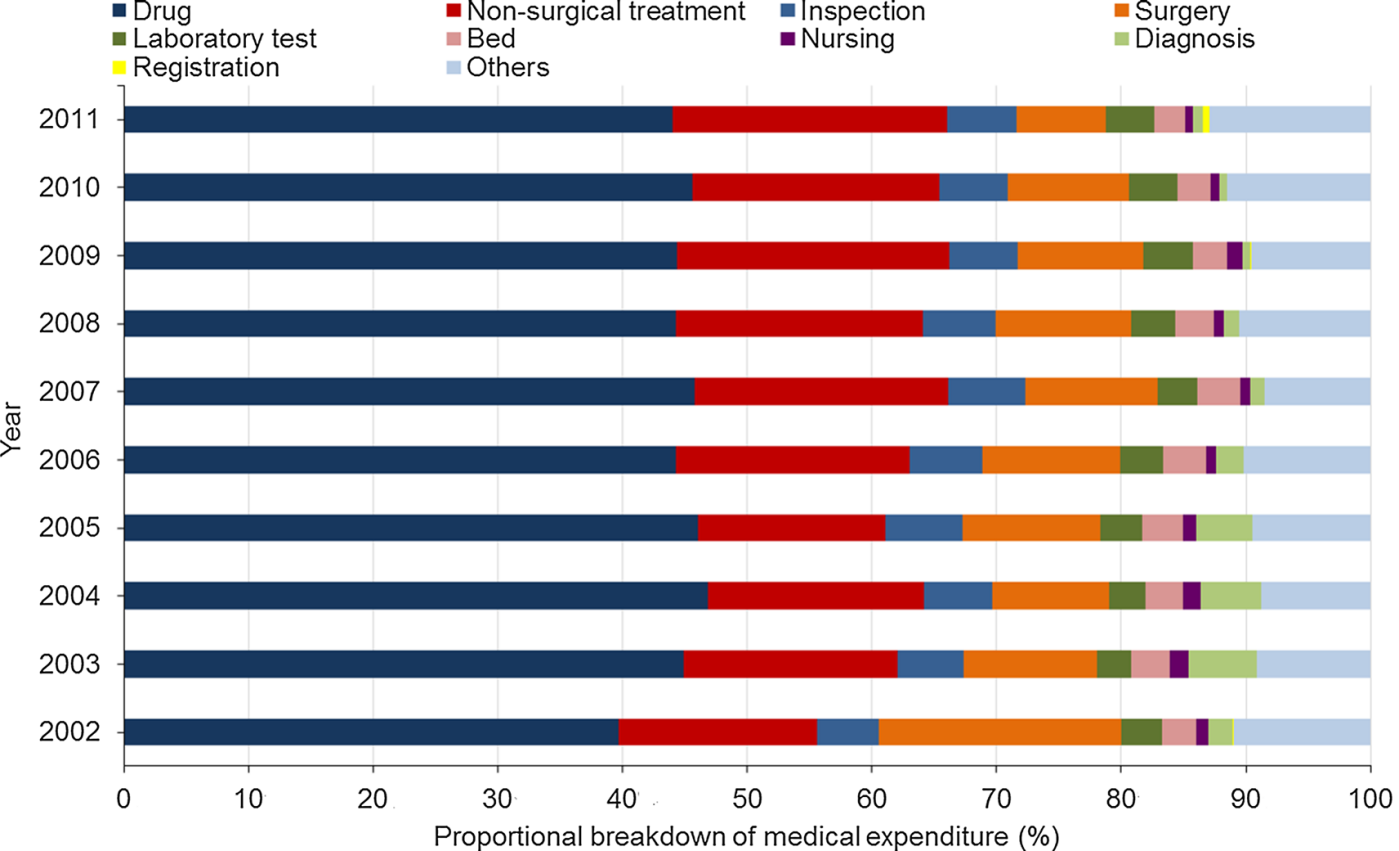
The proportional breakdown of medical expenditures for esophageal cancer diagnosis and treatment per patient. Others include the expenditures for oxygen, blood infusion, heating, and so on

Within the overall average expenditure, drug fee accounted for the largest proportion (45.02%, and never less than 40% since year 2003), followed by non-surgical treatment (19.25%), surgery (10.10%), inspection (5.69%), laboratory test (3.57%), and beds (2.94%). The registration fee accounted for the smallest proportion (0.70%). The proportion of drug fee significantly increased from 39.69% in 2002 to 44.00% in 2011, and reached the highest at 46.84% in 2004. In contrast, the proportion of surgery fee dramatically decreased from 19.47% in 2002 to 7.12% in 2011 (Fig. 4).

## Discussion

In the present study, we found that the overall average expenditure per patient for esophageal cancer increased by 6.27% per year from 2002 to 2011, with an estimate of 38,666 CNY (95% CI 38,178–39,155 CNY) during 2002–2011. Drugs accounted for the largest proportion of the overall expenditure per patient.

The first year after cancer diagnosis is a period with particularly high expenses and intensive treatment [8, 9]. We found that more than 90% of the expenses occurred within 12 months, which was higher than China’s GDP per capita (36,018 CNY in 2011) [10]. As a comparison, the newly diagnosed medical expenditure was less than 40% of the GDP per capita (2005) in the United Kingdom [11], less than 60% (2008–2010) in the United States [12], and less than 65% (1997–2007) in Canada [13]. According to data from China on the average family population (2.9) and the per capita disposable income (21,810 CNY) of urban households in 2011 [10], we found that the average medical expenditure per patient for esophageal cancer was 72.9% of the average family annual income for urban households, indicating that the economic burden imposed by esophageal cancer was relatively high for individual patients’ families in China, especially for low-income families.

Our finding that the medical expenditures in the eastern and central regions were higher than those in the western region mirrors the heterogeneity in economic development among the three regions in China. However, the overall average expenditure per esophageal cancer patient in the Xinjiang province is an exception, which was estimated at 52,167 CNY. The economic burden of esophageal cancer in Xinjiang province is unusually high [6]. Further analysis indicated that esophageal cancer patients in Xinjiang province had more clinical visits than those in other provinces/municipalities. The medical expenditures per esophageal cancer patient significantly differed between the provinces/municipalities; there was a threefold gap between the highest (Guangdong in the eastern region) and lowest (Gansu in the western region) expenditure regions. This result was in agreement with other expenditure studies on esophageal cancer in China [14, 15].

Our finding of an expenditure increase of 1.84 times observed over 10 years is consistent with the results of some other studies. For example, Hu et al. [16] analyzed hospitalization expenses of 1819 patients with esophageal cancer in a cancer hospital in the Shanxi province and found that the adjusted average expenditure per patient increased from 8022 CNY in 2002 to 33,723 CNY in 2011. The time trend of expenditures was almost the same as the time trend of the length of hospitalization stay, suggesting that the length of stay was an important factor affecting the overall average expenditure per patient. Therefore, controlling the length of hospitalization stay has been considered an effective measure for reducing medical expenditures. For this reason, some hospitals try to shorten the hospitalization stays of esophageal cancer patients through implementing clinical pathways, promoting daytime chemotherapy, and other approaches. The two peaks of medical expenditures observed in 2004 and 2008 in the present study might be attributed to the increase in clinical visits and hospitalization stay.

In the present study, we compared the time trend of medical expenditures for esophageal cancer among various subgroups. The expenditures were higher in specialized hospitals than in general hospitals and higher in 3A hospitals than in 3A less hospitals, which might be attributed to the more standardized diagnosis and treatment in specialized and 3A hospitals. The average expenditure per patient increased with the number of clinical visits. There were minor differences in the expenditures among age groups at diagnosis. Medical expenditures were lower for those diagnosed at 65 years and older than for younger patients. This was consistent with results from some other studies, such as a study in the United States that reported the overall expenditures among early-stage esophageal cancer patients older than 65 years of age are, on average, 20% lower than those of younger patients [17]. This is probably because older patients have a smaller probability of cure, and physical or economic pressures forced the patients to abandon intensive treatment or to choose palliative care [18]. ESCC is the most common type of esophageal cancer in China [19]. The higher expenditure for ESCC than for other pathologic types was probably because of the availability of more therapy options and standardized procedures for ESCC. The average expenditure per esophageal cancer patient widely differed between therapy subgroups in the present study. The chemotherapy fee grew fastest, which was probably because of newly developed, expensive anticancer drugs. The expenditure of surgery were higher than those of chemotherapy or radiotherapy, which was probably because surgery is expensive and carries corresponding higher fees for diagnosis and inspection.

We found that the expenditure for stage II–III diseases was relatively higher than those for stage I and IV diseases, which seems to be reasonable if we take the differences of both survival and treatment among patients with I–IV diseases into consideration. As we all know, patients with stage I disease were mainly cured by surgery alone, which was relatively cheap, and those with stage IV disease was commonly treated with palliative care and with short survival period, whereas for patients with stage II and III diseases, multidisciplinary treatment were often adopted, which usually contain expensive antitumor drugs [20]. In addition, in the present study, the proportion of selected patients with stage I disease (7.56%) was lower than the target proportion (25%–30%), even after substantial effort, indicating that most esophageal cancer patients were diagnosed with a late stage disease in the absence of routine esophageal cancer screening or early detection and treatment. A cancer screening program may help with the early diagnosis and treatment of esophageal cancer, which could decrease medical expenditure [21].

Our data showed that drug fee was the largest component of medical expenditure and had never been less than 40% since 2003, although China’s health care reform (initiated in 2009) has planned to reduce the prices of drugs on essential drug lists [22]. The drug fee covers the cost of all the medicine used during the treatment, such as anticancer drugs and supportive drugs for symptoms, most of which are expensive joint venture or imported drugs. Additionally, esophageal cancer patients undergo multiple courses of chemotherapy, resulting in a high drug fee. Therefore, reducing drug fee is important for reducing the financial burden of esophageal cancer patients. In addition, the future policy on drug price addition and economic incentives in medical institutions may increase physician-induced demand, increasing the use of drugs and high-tech inspections [23]. In contrast, the proportions of diagnosis and nursing fees were very low, accounting for less than 3% of the overall medical expenditure, which also had a declining trend from 2002 to 2011. Overall, the expenditure proportion reflecting medical workers’ labor value was still relatively low in the diagnosis and treatment of esophageal cancer. Adjusting charges for medical services to appropriately reflect the value of medical staff services, reduce physician-induced demand, and decrease medical expenditures is very important.

The current analysis has several limitations. First, the gender-specific and stage-specific sample sizes were controlled at the investigation phase, inducing selection bias and potentially decreasing the generalizability of the data. Second, this hospital-based survey only abstracted the expenditure data within the study hospitals; however, some patients also received diagnoses and treatments from other hospitals, suggesting that our data probably under-estimated the economic burden of esophageal cancer. Further follow-up studies are needed to overcome this limitation. Third, in this study, we did not obtain insurance reimbursement information, making it impossible to analyze the actual economic burdens from out-of-pocket payments among esophageal cancer patients during 2002–2011. Finally, this analysis only reported medical expenditure data and did not include non-medical expenditure, which is another important component of expenditure estimation from a patient’s perspective. The non-medical expenditure will be separately reported by the health economic evaluation group of the CanSPUC program using data obtained from face-to-face interviews with esophageal cancer patients.

In conclusion, this study showed that medical expenditure for esophageal cancer in China was burdensome and substantially increased during 2002–2011. To dramatically decrease the economic burden on esophageal cancer patients, solutions are required to reduce personal payment ratio and to reduce medical expenditures incurred by services that are not covered by medical insurance. Our future analysis will use a model-based approach to perform a more aggressive estimate of the medical expenditure per esophageal cancer patient while considering variations, such as the numbers of clinical visits/admissions reported by individual study sites.

## Abbreviations

CanSPUC: Cancer Screening Program in Urban China
CAMS: Chinese Academy of Medical Sciences
EMR: electronic medical record
CNY: Chinese Yuan
GDP: gross domestic product
CPI: consumer price index
ESCC: esophageal squamous cell carcinoma

## References

[1] Ferlay J, Soerjomataram I, Ervik M, Dikshit R, Eser S, Mathers C, et al. GLOBOCAN 2012 v1.0, cancer incidence and mortality worldwide: IARC CancerBase No. 11. Lyon Fr Int Agency Res Cancer. 2013. http://globocan.iarc.fr. Accessed 12 Feb 2016.

[2] Chen WQ, Zheng RS, Zeng HM, Zhang SW (2016). The incidence and mortality of major cancers in China, 2012. Chin J Cancer.

[3] Wei WQ, Yang J, Zhang SW, Chen WQ, Qiao YL (2010). Analysis of the esophageal cancer mortality in 2004–2005 and its trends during last 30 years in China. Chin J Prevent Med.

[4] Zhang SW, Zhang M, Li GL, Wei WQ, Meng FS, Liu ZC (2012). An analysis of incidence and mortality of esophageal cancer in China, 2003–2007. China Cancer.

[5] Dai M, Shi JF, Li N (2013). The design and expected goal for cancer screening program in urban China. Chin J Prev Med..

[6] Ministry of Health of the People ‘s Republic of China. China Health statistics yearbook 2012. Beijing: China Union Medical University Press; 2012. http://www.moh.gov.cn/htmlfiles/zwgkzt/ptjnj/year2012/index2012.html. Accessed 20 Mar 2017 **(in Chinese)**.

[7] Guo LW, Shi CL, Huang HY, Wang L, Yue XP, Liu SZ (2017). Economic burden of esophageal cancer in China from 1996 to 2015: a systematic review. Chin J Epidemiol..

[8] Yabroff KR, Lamont EB, Mariotto A, Warren JL, Topor M, Meekins A (2008). Cost of care for elderly cancer patients in the United States. J Natl Cancer Inst.

[9] Warren JL, Yabroff KR, Meekins A, Topor M, Lamont EB, Brown ML (2008). Evaluation of trends in the cost of initial cancer treatment. J Natl Cancer Inst.

[10] National Bureau of Statistics of China. China statistical yearbook 2012. Beijing: China Statistics Press; 2012. http://www.stats.gov.cn/tjsj/ndsj/2012/indexch.htm. Accessed 20 Mar 2017 **(in Chinese)**.

[11] Agus AM, Kinnear H, O’Neill C, McDowell C, Crealey GE, Gavin A (2013). Description and predictors of hospital costs of oesophageal cancer during the first year following diagnosis in Northern Ireland. Eur J Cancer Care (Engl)..

[12] Guy GP, Ekwueme DU, Yabroff KR, Dowling EC, Li C, Rodriguez JL (2013). Economic burden of cancer survivorship among adults in the United States. J Clin Oncol.

[13] de Oliveira C, Bremner KE, Pataky R, Gunraj N, Chan K, Peacock S (2013). Understanding the costs of cancer care before and after diagnosis for the 21 most common cancers in Ontario: a population-based descriptive study. CMAJ Open.

[14] Yang J, Wei WQ, Niu J, He YT, Liu ZC, Song GH (2011). Estimating the costs of esophageal cancer screening, early diagnosis and treatment in three high risk areas in China. Asian Pac J Cancer Prev.

[15] Ma L, Chen XZ, Liu YQ, Hu XB, Gong HX, Bai YN (2011). The direct economic burden analysis for four common cancer in elderly in Lanzhou city of China. Chin J Gerontol.

[16] Hu ZP, Feng XX, Pan XF (2013). Influence factors of hospitalization expense among esophageal cancer patients. Chin J Public Health.

[17] Mariotto AB, Yabroff KR, Shao Y, Feuer EJ, Brown ML (2011). Projections of the cost of cancer care in the United States: 2010–2020. J Natl Cancer Inst.

[18] Yang M, Xiao J, Chen DX, Gao YX, Lan SY, Zhuang X (2013). Pathway analysis of influencing factors of the hospitalization expense of 8288 esophageal cancer patients. Modern Prevent Med..

[19] Guo LW, Zhang SK, Liu SZ, Chen Q, Zhang M, Quan PL (2016). Human papillomavirus type-18 prevalence in oesophageal cancer in the Chinese population: a meta-analysis. Epidemiol Infect..

[20] Strik C, ten Broek RP, van der Kolk M, van Goor H, Bonenkamp JJ (2015). Health-related quality of life and hospital costs following esophageal resection: a prospective cohort study. World J Surg Oncol..

[21] Gao QY, Fang JY (2015). Early esophageal cancer screening in China. Best Pract Res Clin Gastroenterol.

[22] Yip W, Hsiao W (2014). Harnessing the privatisation of China’s fragmented health-care delivery. Lancet.

[23] Wagstaff A, Yip W, Lindelow M, Hsiao WC (2009). China’s health system and its reform: a review of recent studies. Health Econ.

